# Use of Machine Learning Tools in Evidence Synthesis of Tobacco Use Among Sexual and Gender Diverse Populations: Algorithm Development and Validation

**DOI:** 10.2196/49031

**Published:** 2024-01-24

**Authors:** Shaoying Ma, Shuning Jiang, Olivia Yang, Xuanzhi Zhang, Yu Fu, Yusen Zhang, Aadeeba Kaareen, Meng Ling, Jian Chen, Ce Shang

**Affiliations:** 1 Center for Tobacco Research The Ohio State University Comprehensive Cancer Center Columbus, OH United States; 2 Department of Computer Science and Engineering The Ohio State University Columbus, OH United States

**Keywords:** machine learning, natural language processing, tobacco control, sexual and gender diverse populations, lesbian, gay, bisexual, transgender, queer, LGBTQ+, evidence synthesis

## Abstract

**Background:**

From 2016 to 2021, the volume of peer-reviewed publications related to tobacco has experienced a significant increase. This presents a considerable challenge in efficiently summarizing, synthesizing, and disseminating research findings, especially when it comes to addressing specific target populations, such as the LGBTQ+ (lesbian, gay, bisexual, transgender, queer, intersex, asexual, Two Spirit, and other persons who identify as part of this community) populations.

**Objective:**

In order to expedite evidence synthesis and research gap discoveries, this pilot study has the following three aims: (1) to compile a specialized semantic database for tobacco policy research to extract information from journal article abstracts, (2) to develop natural language processing (NLP) algorithms that comprehend the literature on nicotine and tobacco product use among sexual and gender diverse populations, and (3) to compare the discoveries of the NLP algorithms with an ongoing systematic review of tobacco policy research among LGBTQ+ populations.

**Methods:**

We built a tobacco research domain–specific semantic database using data from 2993 paper abstracts from 4 leading tobacco-specific journals, with enrichment from other publicly available sources. We then trained an NLP model to extract named entities after learning patterns and relationships between words and their context in text, which further enriched the semantic database. Using this iterative process, we extracted and assessed studies relevant to LGBTQ+ tobacco control issues, further comparing our findings with an ongoing systematic review that also focuses on evidence synthesis for this demographic group.

**Results:**

In total, 33 studies were identified as relevant to sexual and gender diverse individuals’ nicotine and tobacco product use. Consistent with the ongoing systematic review, the NLP results showed that there is a scarcity of studies assessing policy impact on this demographic using causal inference methods. In addition, the literature is dominated by US data. We found that the product drawing the most attention in the body of existing research is cigarettes or cigarette smoking and that the number of studies of various age groups is almost evenly distributed between youth or young adults and adults, consistent with the research needs identified by the US health agencies.

**Conclusions:**

Our pilot study serves as a compelling demonstration of the capabilities of NLP tools in expediting the processes of evidence synthesis and the identification of research gaps. While future research is needed to statistically test the NLP tool’s performance, there is potential for NLP tools to fundamentally transform the approach to evidence synthesis.

## Introduction

The use of nicotine or tobacco products is a leading preventable cause of cancer, heart diseases, and lung diseases in the United States [1], with cigarette smoking alone responsible for the death of half a million Americans each year [2]. Notably, sexual and gender diverse individuals, often referred to as the LGBTQ+ (lesbian, gay, bisexual, transgender, queer, intersex, asexual, Two Spirit, and other persons who identify as part of this community) populations, are particularly vulnerable to nicotine and tobacco product use [3]. Both the National Cancer Institute and the Centers for Disease Control and Prevention have recognized the LGBTQ+ populations as a critical target in their efforts to combat tobacco use disparities [4-10].

In response to the pressing need for tobacco control and the rapidly evolving landscape of the tobacco market, the National Institutes of Health (NIH) and other health foundations, including the American Cancer Society, have made substantial investments in tobacco control research and tobacco regulatory science [11,12]. According to our calculations using data from the NIH era reporter, funding for tobacco research has shown a remarkable increase, growing from US $7.7 billion in 2016 to US $11.2 billion in 2021 (Multimedia Appendix 1 [13]). Consequently, the volume of peer-reviewed publications related to tobacco has experienced a significant increase. This presents a considerable challenge in efficiently summarizing, synthesizing, and disseminating research findings, especially when it comes to addressing specific target populations, such as the LGBTQ+ populations.

One promising pathway to rapidly assessing the expanding body of literature is the use of natural language processing (NLP) models. NLP is dedicated to deciphering and comprehending how computers interpret human language, equipping them to analyze extensive data sets of natural language [14-16]. While NLP tools have garnered considerable recognition in biomedical research [4-10], aiding in tasks such as disease surveillance (eg, COVID-19) and diagnosing using medical records [17-23], their potential to expedite near real-time synthesis of evidence in tobacco control research remains untapped [24].

Another gap in existing NLP tools is the lack of applications in synthesizing social science research and modeling. A noteworthy example in the domain of tobacco research is the evaluation of the effectiveness of tobacco control policies, which are often assessed using complex statistical modelling and large-scale survey data. These methods demand a specialized semantic database for labelling studies and interpreting results. However, to the best of knowledge, such a semantic database has not been developed yet. Considering that policy interventions at federal, state, and local levels are designed to reach a large number of populations, the lack of a database to facilitate NLP applications may significantly undermine evidence synthesis and thereby the timely adoption of effective policies [25].

Furthermore, in light of the calls from entities such as the NIH and other health agencies to address tobacco use disparities within priority populations, including LGBTQ+ populations, the development NLP tools to aid in the discovery of effective policies tailored to these special populations remains uncharted territory [26-31]. There is an urgent demand for the development of NLP tools (eg, semantic database, NLP algorithms) in tobacco research that have the abilities to synthesize evidence in social science and assist in research gap discovery for priority populations.

In this pilot study, we aimed to achieve the following goals to address the identified research and application gaps: (1) compile a specialized semantic database for tobacco policy research to extract information from journal article abstracts, (2) develop NLP algorithms that comprehend the literature on nicotine and tobacco product use among sexual and gender diverse populations, and (3) compare the discoveries of the NLP algorithms with an ongoing systematic review of tobacco policy research among LGBTQ+ populations [32]. While this pilot study does not fully address the gaps by developing a comprehensive evidence synthesis or discovery tool for tobacco research, the outcomes may pave the road for future tools that can achieve this goal. Our vision is that NLP tools may be able to assist academic scholars and policy makers in prescribing public health policies, such as tobacco control policies, and addressing public health needs, such as reducing health disparities.

## Methods

### Development of a Tobacco Research Domain–Specific Semantic Database

#### Overview

To generate a tobacco research domain-specific semantic database, we used an iterative process that combines expert opinions and the reading of tobacco research papers in 4 leading tobacco journals (*Tobacco Control*, *Nicotine and Tobacco Research*, *Tobacco Induced Diseases*, and *Tobacco Prevention and Cessation*). The main categories of keywords were the follows: (1) tobacco use behaviors, prevalence, and outcomes; (2) population characteristics; (3) geographic locations; (4) method and inference; (5) policy; (6) tobacco products; (7) relation statement; and (8) tobacco characteristics. Under each main category, there were one or more subcategories, and each subcategory contained a list of named entities. Table 1 presents the categories of named entities in a domain-specific semantic database that were used for training and improving a language model for tobacco research on sexual and gender diverse populations. These categories are based on journal articles’ keywords, further guided by existing literature on how to use NLP methods to synthetize public health evidence [25,33]. These categories are important components of a study, encompassing measures, methods, results, conclusions, and hypothesis testing.

**Table 1:** Main categories and subcategories of named entities.

Main categories	Subcategories
Tobacco use behavioral outcomes	Tobacco cessation Exposure to tobacco-related or antitobacco content, or exposure to secondhand or thirdhand smoking Health and disease Perception and belief Tobacco use prevalence Time period
Population characteristics	Age groups Sex Sexual and gender diverse populations Racial and ethnic minoritized groups Socioeconomic status
Geographic locations	Countries, states, provinces, or cities
Method and inference	Data Methodology Statistics
Policy	Marketing Law, policy, and regulation Regulation body Treatment
Tobacco products	Combustible tobacco products Noncombustible tobacco products
Relation statement	Relation terms
Tobacco characteristics	Chemical Flavor

#### Journal Selection

We chose 4 peer-reviewed tobacco-specific multidisciplinary journals, namely, *Tobacco Control*, *Nicotine and Tobacco Research*, *Tobacco Induced Diseases*, and *Tobacco Prevention and Cessation*, to extract articles and compile keywords at the initial stage. The first 2 are among the journals that have the highest impact factors in addiction research; in 2022, *Tobacco Control* had an impact factor of 5.2 and a 5-year impact factor of 5.7 [34], and *Nicotine and Tobacco Research* had an impact factor of 4.7 and a 5-year impact factor of 4.2 [35]. *Tobacco Induced Diseases* [36] and *Tobacco Prevention and Cessation* [37] are 2 other peer-reviewed journals that specifically publish research on nicotine and tobacco products but are not as highly ranked as the other 2 journals. The textual data from the 4 peer-reviewed journal articles contained a total of 2993 abstracts from published papers from 2015 to early 2021.

While the 2993 articles extracted from these journals do not represent the full body of tobacco research, they cover a significant share of tobacco studies and integrate evidence across the 5 translational research stages: basic research, preclinical research, clinical research, clinical implementation, and public health. These journals also ask authors to specify how the research reported contributes to tobacco control objectives, which have policy implications. Alternatively, a random sampling from PubMed searches using tobacco related terms may not yield studies that are necessarily translational in nature. Therefore, we focused on the articles published in the 4 journals in our study.

#### Iterative Process to Expand Terms (Named Entities) in the Database

The general process included the following iterative steps: (1) to generate initial annotation data, we first complied key terms from extracted articles and allocated key terms to categories using group discussions; (2) we enriched the database using various sources and group discussions (more specific descriptions below); (3) we fine-tuned the *spaCy* en_core_web_lg model with the initial annotation and following iterative versions of data (the en_core_web_lg model is a pretrained large language model that can extract multiple general named entities); (4) we expanded the list of named entities to include more keywords of similar meanings using SeedNER [38,39], that is, a small set of initial labeled examples or patterns that was used as a starting point for training a model; (5) we searched the occurrence of each keyword in the 2993 paper abstracts and kept those with high frequency; (6) during this process, named entities that were too generic to yield meaningful relations were removed from the database; and (7) we repeated steps 3 to 6 until the set of entities reached our satisfaction during group discussions.

Specific approaches were used for conducting step 2. For categories including “tobacco use behavioral outcomes,” “tobacco products,” and “tobacco characteristics,” the iterative process involved four steps: (1) discussions to determine whether to include newly identified key terms and how to allocate them into additional subcategories (Table 1); (2) using a named entity recognition (NER) model to extract named entities from 2993 paper abstracts from the 4 specific journals; (3) randomly sampling and reviewing the output of the NER model, correcting identified errors, and adding missed NERs; and 4) repeating steps 1 to 3 until we were satisfied with the model output.

The categories “population characteristics,” “geographic locations,” and “relation terms” are commonly used concepts in real life and not specific to tobacco control. We used Google searches, Wikipedia, and WordNet to enrich the key terms. In addition, for the “method and inference” category, we used the glossary of an econometrics methodology textbook by Cameron and Trividi to enrich the terms [40]. This textbook is widely used in economics and social science and its glossary should provide sufficient terms for this category.

For the “policy” category, we drew named entities from 2 sources that comprehensively summarize available tobacco control policies in the regulatory space. The first source was a peer-reviewed journal article by McDaniel et al [41] that conducted an intensive policy scan of all possible regulations that can contribute to tobacco endgame. The second source was the World Health Organization’s report on the global progress in implementing tobacco control policies, as recommended by the World Health Organizaiton’s Framework Convention on Tobacco Control [42], which is the largest public health treaty signed by 182 countries and prescribes a comprehensive set of tobacco control policies. These policies are classified into 5 groups: M (monitor tobacco use and prevention policies), P (protect people from tobacco smoke), O (offer help to quit tobacco use), W (warn about the dangers of tobacco), E (enforce bans on tobacco advertising, promotion, and sponsorship), and R (raise taxes on tobacco) [42]. These sources cover policy key terms related to both national and international contexts and together create the most comprehensive policy terms to our knowledge.

### Development of NLP Algorithms That Comprehend the Literature on Nicotine and Tobacco Product Use Among Sexual and Gender Diverse Populations

We used RoBERTa, an optimized BERT (bidirectional encoder representations from transformers)-based language model [43], to perform NER tasks. BERT is a state-of-the-art language model that excels at tasks such as sentiment analysis and text summarization. By learning patterns and relationships between words and their context in text, BERT can extract named entities that it has learned during training and potentially discover new ones.

We developed an NER model based on RoBERTa using the Python (Python Software Foundation) programming language and the *spaCy* library [44]. We began by defining 36 labels of categories (main and subcategories; Table 1) and extracting 1582 named entities using the existing NER model RoBERTa. Next, those named entities were used to tag abstracts and create a training set, using the annotation tool Prodigy [45]. A subset of the abstracts with labeled named entities was reviewed by 2 domain experts to identify key terms that were missing in our semantic database, which were added to the lists of named entities.

The RoBERTa model was then updated based on the richer database and further trained for a maximum of 20,000 steps, with early stopping implemented if no improvement was observed for 1600 consecutive steps. With a series of iterations, we used the updated RoBERTa model to assess the 2993 abstracts and labeled them with the categories.

When identifying studies related to LGBTQ+ populations, it is important to understand that this community is heterogeneous [46,47]. Given that LGBTQ+ key terms are included in the “population characteristics” categories, we were able to identify LGBTQ+ populations based on categorization. There were 111 LGBTQ+-related named entities in our database.

### Comparison of the Discoveries of the NLP Algorithms With an Ongoing Systematic Review of Tobacco Policy Research Among LGBTQ+ Populations

Ideally, we would like to compare the results from our tools with those from systematic reviews and meta-analyses of studies related to tobacco control issues among LGBTQ+ populations. Systematic reviews and meta-analyses are state-of-the-art evidence synthesis methods that can provide the ground truth [48-50]. While we are currently conducting a separate systematic review of the effectiveness of tobacco control policies among LGBTQ+ populations, this review has not been finalized yet [32]. Nonetheless, the ongoing systematic review does provide some data points for comparisons, including the number of studies extracted from the 4 journals and presence of policy assessment. Therefore, we conducted comparisons of these 2 domains.

### Ethical Considerations

This study does not involve human subjects, as it synthesizes data from research articles published at peer-reviewed journals. The Ohio State University Institutional Review Board has determined that it contains no human subjects and thus no further review is needed (study number: 2021E0776).

## Results

In total, we identified 33 articles relevant to sexual and gender diverse populations from the 2993 abstracts. Our trained model successfully extracted 773 named entities (181 unique named entities) from the 33 paper abstracts to describe the themes of these articles. Among the 773 extracted named entities, 688 were already learned by the model during training, while 70 were new time- or age-related words (eg, 18 years, 2013), 9 were new statistical terms (eg, N=20), and 6 were newly discovered and labeled within other categories. We did not observe any newly discovered policy-related terms.

**Figure 1:**
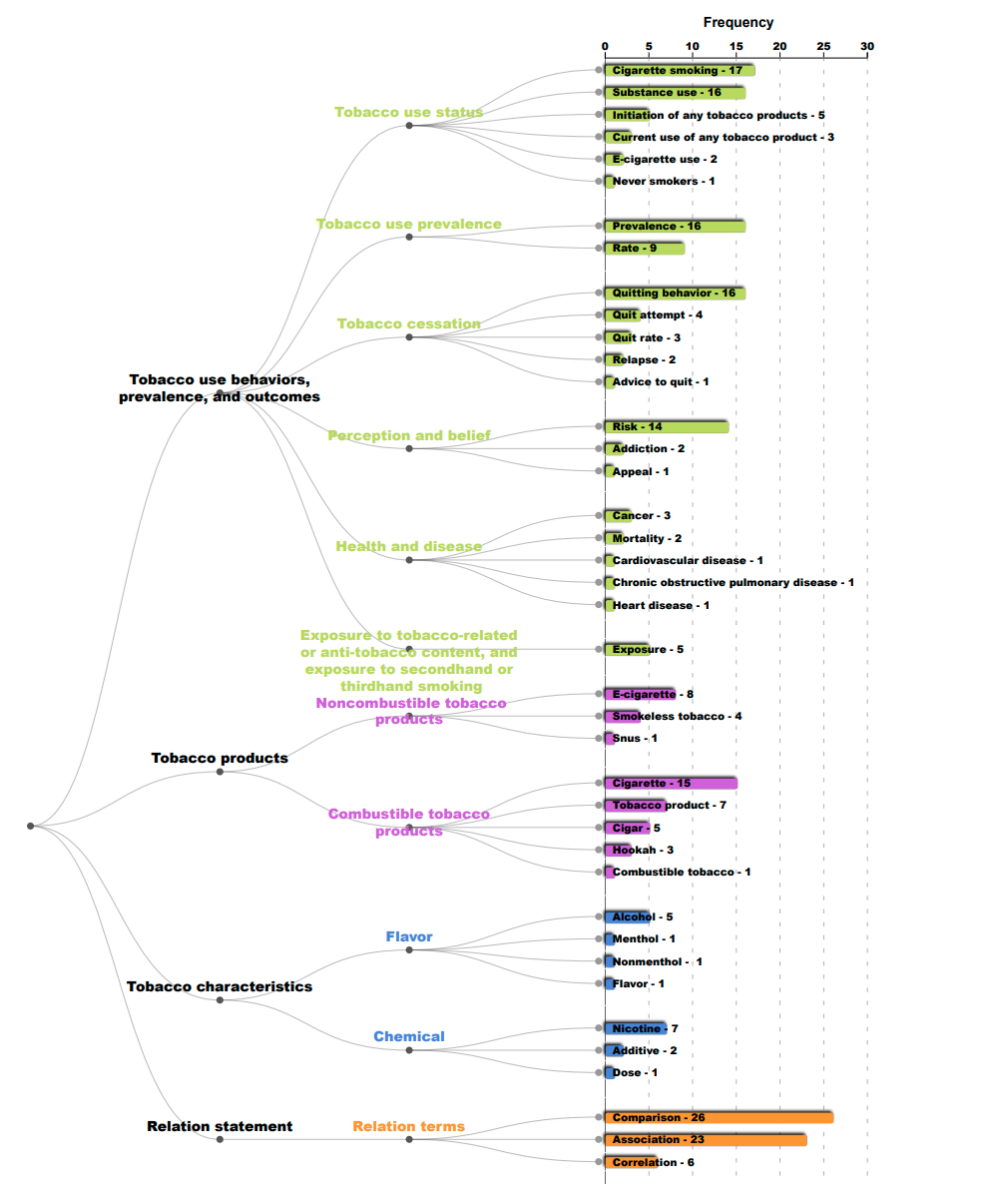
Hierarchy and frequency counts of named entities extracted from published research in tobacco-specific journals from 2015 to early 2021 in 4 main categories: tobacco use, products, characteristics, and relation statement. Numbers represent the frequency of the corresponding named entity by paper abstract.

**Figure 2:**
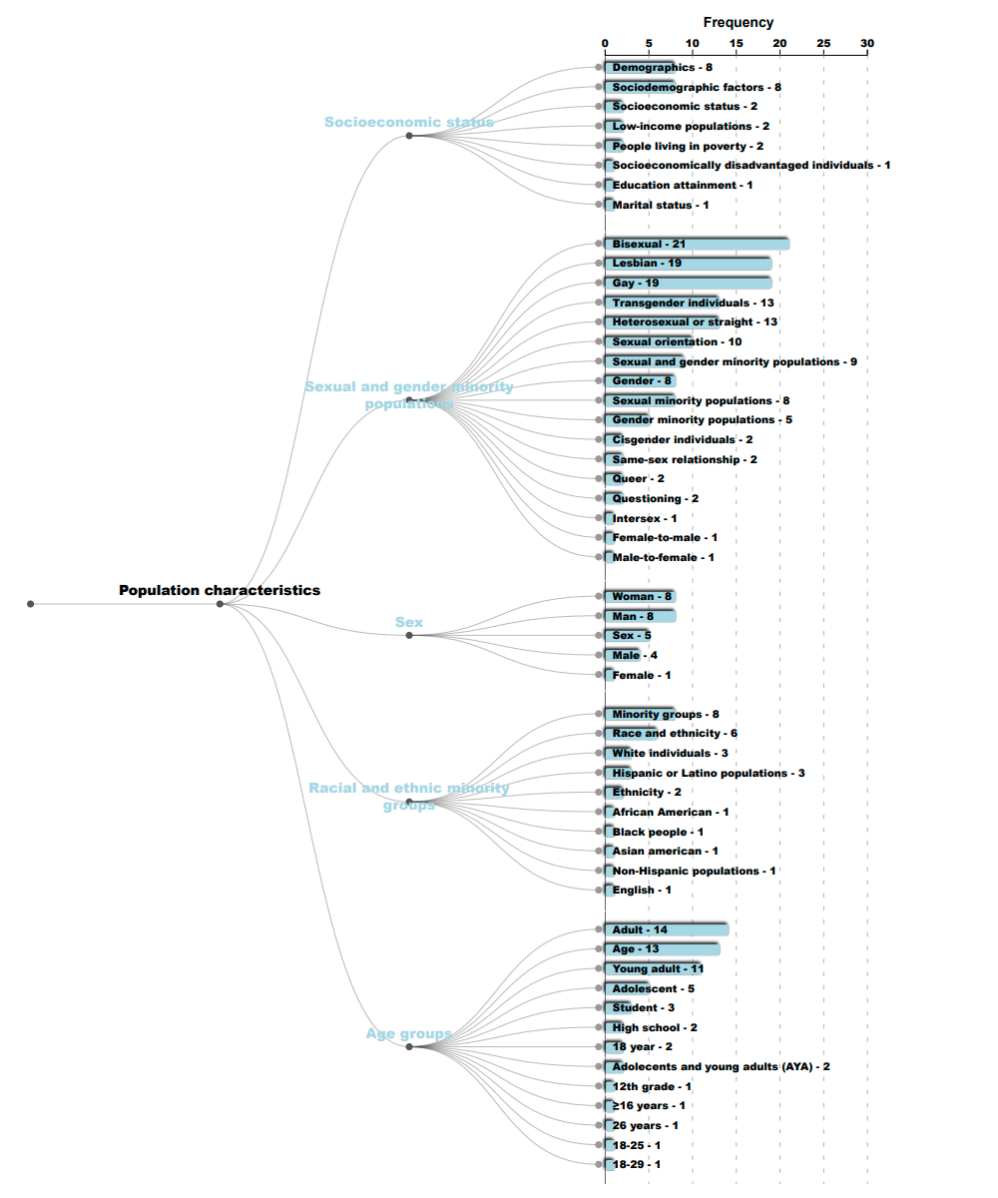
Hierarchy and frequency counts of named entities extracted from published research in tobacco-specific journals from 2015 to early 2021 in the main category of population characteristics. Numbers represent the frequency of the corresponding named entity by paper abstract.

**Figure 3:**
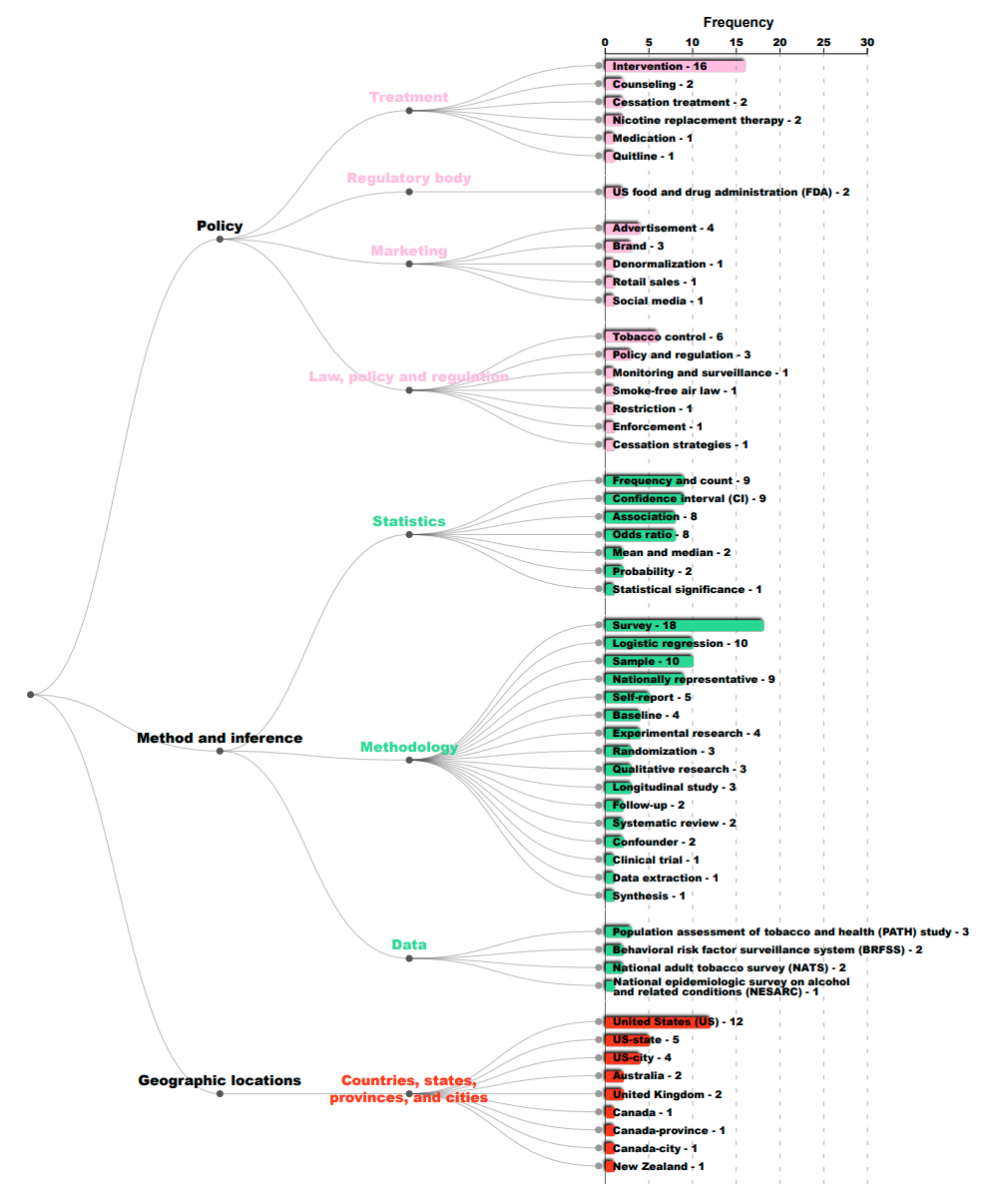
Hierarchy and frequency counts of named entities extracted from published research in tobacco-specific journals from 2015 to early 2021 in 3 main categories: policy, methods and inference, and geographic locations. Numbers represent the frequency of the corresponding named entity by paper abstract.

In Figures 1-3, we present the hierarchy of named entities extracted from abstracts in published papers that studied nicotine or tobacco product use among sexual and gender diverse individuals. Each number on the right is the frequency of the corresponding named entity by paper abstract. Named entities with the same color belong to the same main category.

According to our tool, among the 33 tobacco studies related to LGBTQ+ populations, the most frequent use outcomes were “cigarette smoking” (n=17), “substance use” (n=16), “prevalence” (n=16), and “risk” perception (n=14). Also, for these populations, “cigarettes” (n=15) were the most frequently mentioned combustible tobacco product and “e-cigarettes are” (n=8) was the most frequently mentioned noncombustible tobacco product. In addition, for tobacco characteristics, “alcohol” (n=7) and “nicotine” (n=5) were the most mentioned attributes among LGBTQ+ tobacco research papers.

The relation statement findings suggest that a majority of the studies examined “comparison” (n=26), “association” (n=23), and “correlation” (n=6). We found no studies that explicitly used the term “causal” or “causality” in the studies.

The population characteristics mentioned in the studies illustrated that among socioeconomic status terms, the most frequently included were “demographics” (n=8) and “SES factors” (n=8). Among sex and sexual and gender minority terms, the most frequent ones were “bisexual” (n=21), “lesbian” (n=19), and “gay” (n=19). Among racial and ethnic minority group terms, the most frequent ones were “minority groups” (n=8) and “Race/ethnicity” (n=6). For age group terms, the terms included “adult” (n=14), “young adult” (n=11), “adolescent” (n=5), “students” (n=3), and “adolescents and young adults” (n=2).

The policy category showed that in these studies, the most mentioned term was “intervention” (n=16). In addition, while the general term “tobacco control” was mentioned in 6 studies, only 1 study contained any specific policy term (“smoke free air law”). As such, there was a significant gap in policy research among the published articles in the 4 leading tobacco journals between 2015 and early 2021, since only 1 study mentions specific policies when it comes to tobacco research among the LGBTQ+ populations. The statistics and methodology terms further indicated that the most used terms included “survey” (n=18) and “logistic regression” (n=10), and relatively fewer studies mentioned terms related to causal inferences, such as “experimental research” (n=4), “randomization” (n=3), and “clinical trial” (n=1). The studies mentioning “US” also dominated in the numbers, with 12 studies in total. Several studies that assessed countries with multilevel governing levels, such as Canada and the United States, also appeared to have mentioned “state,” “city,” and “province,” suggesting that attention was paid to these defined areas.

We next compared our results using the NLP tools with our ongoing systematic review. Similar to the conclusions of the ongoing systematic review, we found very few studies that yielded specific policy recommendations. This finding was further corroborated by the lack of causal inference methods labeled by the NLP tool. While our NLP tool cannot replace systematic reviews just yet, it does show potential to complement the existing methods and requires less human supervision (systematic reviews usually require at least 2 human coders).

## Discussion

This pilot study builds a semantic database dedicated to tobacco research and developed NLP algorithms to automatically identify, extract, and summarize textual data from published tobacco studies. We further demonstrated a user case wherein we assessed LGBTQ+ tobacco research by labeling key components of a tobacco study: tobacco use outcomes, tobacco characteristics, population characteristics, geographic locations, method and inference, and policy relevance.

It is worth noting that the components we categorized, such as “method and inference,” align with the typical sections found in scientific articles in social science, including measures, methods, results, conclusions, and hypothesis testing. As a result, our tool extracts text segments that are frequently assessed in evidence synthesis, thereby showing the potential of using NLP tools to enhance systematic reviews and facilitate meta-analyses [25].

Additionally, we leveraged the NLP algorithms we created to identify gaps in tobacco research concerning the LGBTQ+ populations and concluded that there is a scarcity of studies assessing policy impacts on this demographic using causal inference methods. This finding is consistent with our ongoing systematic review [32], highlighting how NLPs have the capacity to aid in both evidence synthesis and research gap discoveries. This, in turn, has the potential to streamline research efforts, reduce labor costs, and influence the trajectories of future research directions [51,52].

Using the NLP tool, we further found some interesting patterns in tobacco research involving LGBTQ+ populations. It appears that the product drawing the most attention in the field is cigarettes or cigarette smoking and that the number of studies of various age groups is almost evenly distributed between youth or young adults and adults. Moreover, the existing evidence body is dominated by studies coming from the United States. These patterns are consistent with the research needs to reduce cigarette smoking among LGBTQ+ populations in the United States, where 16.1% of LGBTQ+ adults and 17.4% of LGBTQ+ high schooler students smoke cigarettes—this is 4% to 6% higher than their heterosexual counterparts [53,54]. Therefore, our findings align with the ongoing research needs and the financial investments made by the US health agencies like the NIH, thereby bolstering the confidence in the NLP tool that we developed.

Finally, while the semantic database and language model in this pilot study are designed to extract and summarize key components of tobacco research, many of the terms and labeling categories are broad and applicable to public health and social science research in general, such as “methods and inference” and “relation terms.” Therefore, our tool has the potential to transform the evidence synthesis paradigm in tobacco control and public health at large by enabling more efficient and effective analyses of large volumes of textual data. Future tool development may extend its reach to other public health domains, fostering the real-time translation of research findings into evidence-based policymaking, thereby contributing significantly to the advancement of public health initiatives.

Our study has several limitations. First, for the development of keywords and the application of the NLP, we focused on 4 peer-reviewed tobacco-specific research journals, which were not representative of the entire tobacco control literature. However, considering the prominence and extensive content covered by these journals, we believe that this selection is unlikely to introduce significant selection bias or result in the omission of crucial keywords. Second, although we used our ongoing systematic review as a benchmark for the qualitative assessment of the results obtained in this pilot study, we did not perform a quantitative comparison of our findings with the ground truth derived from the systematic review. This quantitative evaluation, which might include measures like Cohen kappa, was not conducted because the systematic review has not yet been finalized. Consequently, future research endeavors are required to undertake a thorough quantitative comparison between the training data and the established ground truth using statistical testing for a more comprehensive assessment of the NLP tool’s performance.

Despite the limitations, our pilot study serves as a compelling demonstration of the capabilities of NLP tools in expediting the processes of evidence synthesis and the identification of research gaps. Expanding the scope of this pilot research to encompass other public health disciplines, extending beyond the realm of tobacco control, holds the promise of fundamentally transforming the approach to evidence synthesis. Such expansion has the potential to play a pivotal role in shaping policy development across a wide spectrum of public health domains.

## Appendix

Multimedia Appendix 1 Tobacco-related funding from the National Institutes of Health (NIH), 2010-2022. Data was obtained from the National Institutes of Health [13].

## Abbreviations

BERT: bidirectional encoder representations from transformers
LGBTQ+: lesbian, gay, bisexual, transgender, queer, intersex, asexual, Two Spirit, and other persons who identify as part of this community
NER: named entity recognition
NIH: National Institutes of Health
NLP: natural language processing

## Data Availability

The data sets generated and analyzed during this study are available in the GitHub repository [55].
