# Supplementary figures and images for: Use of Machine Learning Tools in Evidence Synthesis of Tobacco Use Among Sexual and Gender Diverse Populations: Algorithm Development and Validation
Source: JMIR Form Res. 2024 Jan 24;8:e49031. doi: 10.2196/49031 (PMC10851114; doi:10.2196/49031)

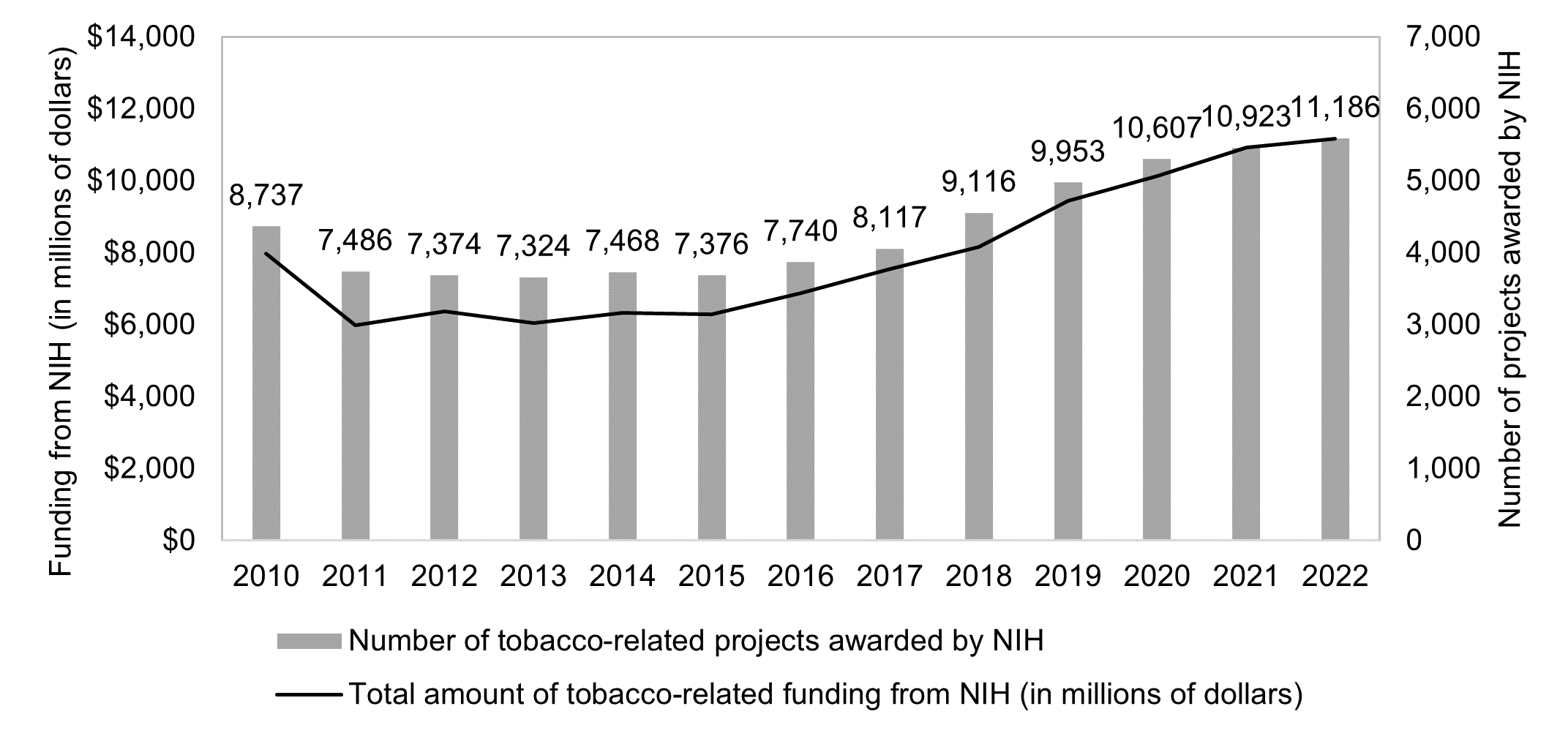

Supplement: Multimedia Appendix 1 [file formative_v8i1e49031_app1.png]
